# Depression and Creativity During COVID-19: Psychological Resilience as a Mediator and Deliberate Rumination as a Moderator

**DOI:** 10.3389/fpsyg.2021.665961

**Published:** 2021-05-06

**Authors:** Yanhua Xu, Jinlian Shao, Wei Zeng, Xingrou Wu, Dongtao Huang, Yuqing Zeng, Jiamin Wu

**Affiliations:** ^1^College of Resource Environment and Tourism, Capital Normal University, Beijing, China; ^2^School of Geography, South China Normal University, Guangzhou, China

**Keywords:** depression, creativity, psychological resilience, deliberate rumination, moderated mediation model, college students

## Abstract

**Purpose:** The pandemic of coronavirus disease 2019 (COVID-19), which has had a significant impact on people’s lives, has apparently increased the incidence of depression. Although the topic of how depression affects creativity is contested, previous research has revealed a significant relationship between the two. The purpose of this study is to further investigate the relationship and the mechanisms that operate between depression and creativity.

**Methods:** A total of 881 students at an independent college in China completed a questionnaire consisting of the Self-Reported Depression Scale, Runco Ideational Behavior Scale, Psychological Resilience Scale, Deliberate Rumination Scale and demographic information. Among the respondents, 317 (36.0%) were male and 564 (64.0%) were female, all of whom were from the same grade. Correlation analyses were conducted, and then the researchers carried out mediation analysis and developed a moderated mediation model.

**Results:** The results indicated that (a) depression was positively related to creativity (*r* = 0.085, *p* < 0.05); (b) psychological resilience mediated the relationship between depression and creativity; specifically, psychological resilience was negatively related to depression (*r* = −0.462, *p* < 0.01), which in turn was positively related to creativity (*r* = 0.198, *p* < 0.01); and (c) deliberate rumination moderated the relationship between depression and psychological resilience, showing a significant negative correlation with depression (*r* = 0.138, *p* < 0.01), psychological resilience (*r* = 0.078, *p* < 0.05), and creativity (*r* = 0.288, *p* < 0.05); specifically, higher levels of deliberate rumination strengthened the negative correlation between psychological resilience and depression.

**Conclusion:** The results suggest that depression is a positive predictor of creativity and may promote creativity to some extent. Further, individuals with greater psychological resilience are more creative than those with less psychological resilience, as it is a question of whether they can and to what extent they can effectively use depression as an emotional resource. Last, an individual’s level of deliberate rumination moderates the mediating process, especially at the stage where depression is associated with psychological resilience. These findings advance understanding of the mechanisms that operate between depression and creativity.

## Introduction

The coronavirus disease 2019 (COVID-19) has had an increasing impact on humans. According to the World Health Organization, COVID-19 may persist for a long time. As the pandemic has worsened, governments have locked downed cities and decreed social-isolation measures for residents (Zhou et al., 2020), which has increased the risk of people developing psychological disorders. A survey of 1,770 Chinese citizens showed a 47.1% prevalence of depression and an 18.2% prevalence of major depressive symptoms due to social isolation and other reasons (Ran et al., 2020). Similarly, a longitudinal investigation showed that the stress of the pandemic has had a significant impact on the college students (Wang et al., 2020), who showed varying degrees of anxiety and depression due to factors such as financial pressure and academic delays (Dhar et al., 2020). Therefore, it is important to focus on the emotional impact of the pandemic on people.

This public health emergency has triggered significant negative emotions in individuals, especially depressive symptoms. Social isolation can cause loneliness and interpersonal disconnection. Especially for those who are socially most vulnerable, feelings of exhaustion, anxiety, distress, and trauma can grow, leading to significant clinical symptoms of psychological disorders and an increased risk of depression (Pietrabissa and Simpson, 2020). Stressors such as the economic crisis, the threat of unemployment or the fear of losing a loved one can further increase the psycho-emotional risk of fatigue, depression, insomnia, and loneliness (Bartoszek et al., 2020). Also, though they respond differently depending on their developmental stage, children may experience high rates of anxiety, depression, and post-traumatic symptoms (Marques de Miranda et al., 2020). Thus, depression has been identified as a main psychological disorder emerging in individuals during the pandemic, affecting their lives and mental health.

Though depression may negatively affect individuals, its relationship with creativity has been of interest to scholars (Kim et al., 2016). Being in a negative frame of mind does not always have a negative impact on a person’s motivation and creativity. It has been suggested that individuals exhibit great artistic creativity when they are physiologically susceptible to negative emotions and exposed to environments that bring about strong negative emotions (Akinola and Mendes, 2008). However, the relationship between psychopathology and creativity has long been controversial (Paek et al., 2016), and more in-depth research is needed.

Depression, as a negative emotion, is closely related to an individual’s mental health and may affect an individual’s psychological resilience (Poole et al., 2017; Liu et al., 2018; Xiang et al., 2018, 2020), but there has been little research on psychological resilience as a mediating pathway for the transformation of depression into creativity. Similarly, it has been shown that the relationship between negative emotions and creativity may be influenced by an individual’s internal psychological environment, such as their level of rumination (Verhaeghen et al., 2005; Cohen and Ferrari, 2010). What is more, rumination has served as a mediator and moderator in studies related to negative emotions such as anxiety and depression (Liu et al., 2017). However, there is a paucity of research on how rumination is associated with depression, psychological resilience and creativity. This has prompted the authors to reflect on how depression may have affected an individual’s creativity during the pandemic and whether psychological resilience and rumination played a role in this relationship.

In short, the role of psychological resilience and rumination in mediating the relationship between depression and creativity during COVID-19 has rarely been studied. Further research is warranted as it may be relevant to the mental health of individuals during the pandemic as well as to their social adjustment and creativity in the post-pandemic era.

This study explores the relationship between depression and creativity among college students during COVID-19 as well as the mediating role of Psychological Resilience and considers the inclusion of deliberate rumination as a moderator in the model. These four variables and their relationships are discussed in the following section.

## Theoretical Basis and Hypotheses

### Creativity

Creativity has been a subject of extensive and continuing discussion. Defined as the process by which people think and act to produce novel works (Guilford, 1950), creativity was said to comprise four steps: preparation, incubation, illumination, and verification (Wallas, 1926). After decades of extensive research, a deeper understanding developed of the competencies, characteristics, and cognitive processes associated with creativity (Plucker et al., 2004; Cadle, 2015), and researchers took a more nuanced and interdisciplinary approach to understanding creativity (Goswami, 1996). For example, a recent grounded-theory study proposed that the creative process for journalists goes through four stages: cognition, cultivation, capture, and reflection (Rubenstein et al., 2017). Some studies have taken a dynamic view, arguing that creative achievement and creative uncertainty are key to the creative process (Corazza, 2016). Some researchers have classified creativity as “big-C” or “little-c” (Gardner, 1993). Big-C creativity refers to excellence in fields such as art, science and other fields, while “little-c” creativity refers to the ability to problem-solve so as to adapt to changes in society and daily life and create new opportunities for oneself (Runco, 2004).

A wealth of research has provided a broader understanding of the meaning and process of creativity, but no one definition is universally accepted by scholars. However, it is generally accepted that creativity is a cognitive process characterized by the ability to generate novel ideas (Runco and Basadur, 1990; Varshney et al., 2013). It is closely related to intelligence (Sternberg, 2017), including to important competencies such as divergent thinking (Zhu et al., 2019), problem identification (Hah, 2006), creative problem-solving (Isen et al., 1987), and breadth of attention (Fredrickson and Branigan, 2005). Therefore, it can be inferred that creativity is influenced by a very large number of factors. According to Sternberg’s triangular theory of creativity, individuals challenge the crowd, themselves and the zeitgeist in different combinations that lead to different manifestations of creativity (Sternberg, 2018). What is more, creativity behaves differently in different situations, such as in artistic creation (Xurui et al., 2018), corporate innovation (Lee et al., 2004; Ding et al., 2019), everyday emotion (Han et al., 2019), and psychopathology (Sampedro et al., 2020). But the essence of creativity can be seen as a cognitive process (Leschziner and Brett, 2019).

Much research has examined how creativity as a cognitive process is influenced by other factors, and the relationship between emotion and creativity has been of great concern. The mainstream consensus is that positive emotions promote creativity, but research on the relationship between negative emotions and creativity is somewhat contradictory. A comparative analysis found that under induced conditions negative emotions had a more obvious effect on creative problem-solving than did positive emotions (Kaufmann and Vosburg, 1997). Conversely, it has also been argued that negative emotional states take a bottom-up cognitive processing and that being overly cautious, which decreases attention span, diminishes an individual’s creative expression (Miller et al., 2019; Lai et al., 2020). In the dual-path model of creativity, it is argued that being in activating mood states, whether positive or negative, enhances creativity, and it is just that positive emotions stimulate cognitive flexibility, while negative emotions stimulate persistence (De Dreu et al., 2008). Thus, a direct linear relationship between negative emotions and creativity has not been confirmed by a strong and accepted research result. Also, the relationship between different dimensions of negative emotions, especially of psychological disorders, and creativity has not been definitively established (Kim et al., 2016).

Based on the above studies, we can affirm that the nature of the correlation of negative emotions (such as anxiety, depression and suppression) and creativity is contested.

### Depression

Depression, as a major factor in negative affect, is seen as one manifestation of psychological disorder (Beck et al., 2003; Cox et al., 2019). Major features of depression are considered to include diminished self-esteem and self-motivation and ineffective perception of important life goals (Lovibond and Lovibond, 1995; Antony et al., 1998). When it comes to creativity and depression, there is a saying that “there is a thin line between genius and madness,” and many studies have shown a link between the two. In his studies of luminaries, Post (1996, 2018) found that while these famous people had great character qualities and creative ability, they also showed more frequent “neuroticism” and a higher tendency toward depression and alcoholism than the average person, especially those who were writers and visual artists. Bipolar depression has also been associated with increased creativity (Andreasen, 1987; Richards et al., 1988), and people with such a disorder are more likely to have creative occupations (Kyaga et al., 2018). Further support can be found in several meta-analyses in which mood disorders (anxiety, depression, and ADHD) and psychiatric disorders were more strongly associated with the creativity of geniuses (big-C) than with the creativity of the general population (little-c) (Acar and Sen, 2013; Paek et al., 2016). Importantly, a study in which adrenal steroids were used as a measure of depressive mood found that individuals who are physiologically predisposed to negative emotions are more creative if exposed to a negative emotional environment (Akinola and Mendes, 2008), providing a biological evidence for a link between depression and creativity. What is more, some studies have suggested that emotions may influence the encoding of information and the storage of memories from the outset (Plass and Kalyuga, 2019). In addition to seeing depression as an influential factor independent of creativity, depressive mood is part of informational functioning signal when individuals dealing with creative tasks (Baas et al., 2008). In contrast to positive emotions, which facilitates creative performance on funny tasks, negative mood helps individuals to achieve better creative performance on serious tasks, pushing individuals to work harder to meet standards (Ronald et al., 2007).

In summary, it is reasonable to hypothesize that:

H1 Depression is positively correlated with creativity.

While many studies have shown a link between depression and creativity, others have shown that the direct effect of depression on creativity is not significant (Silvia and Kimbrel, 2010), suggesting that no consistent and systematic conclusion exists about the internal mechanisms underlying the relationship between depression and creativity. Enhanced exploration of the mediation effect and moderate effect between depression and creativity may help uncover the relationship between the two.

### Psychological Resilience

In the field of developmental psychology, psychological resilience refers to the ability of individuals to demonstrate better physical fitness, response and coping skills despite experiencing events that affect them greatly (Rutter, 2006; Huisman et al., 2017). Resilience enables individuals to find positive meaning in stress, to withdraw from negative emotions, to adapt to changing external stressors and to bounce back from the experience (Tugade and Fredrickson, 2004), which involves personal emotion and cognitive processes (Block and Kremen, 1996; Tugade and Fredrickson, 2004). Psychological resilience can be categorized as individual resilience or collective resilience (Rutter, 1999). From a behavioral perspective, psychological resilience can be viewed as a set of behaviors or attitudes and skills that enable effective coping with depression and stress (Sharpley et al., 2011, 2012). It can also be viewed as a stable personality trait that can influence emotion regulation processes (Masten, 2007; Hou and Ng, 2014). These multiple definitions reveal psychological resilience to be a multi-dimensional human ability (Dunn and Predescu, 2019) to adapt, recover and bounce back from adversity, stressful situations, threats or losses and to stimulate post-traumatic growth (van der Werff et al., 2013). The Rutter Model of Development suggests that there are four mechanisms by which mental resilience functions: (1) reducing the negative effects of risk factors; (2) minimizing collateral reactions to stressful events; (3) enhancing self-esteem and self-efficacy; and (4) providing opportunities and resources for individuals (Rutter, 1999).

Evidences suggest that psychological resilience plays a significant role in the regulation of negative emotions, helping people to emerge from emotional disturbances (e.g., depression, anxiety) and enhancing self-growth. When a stressor is present, an individual’s psychological resilience can prevent psychological distress: for example, breast cancer patients acquired the resilience to cope with psychological problems that arise during treatment and effectively improved their quality of life (Sharpley et al., 2014; Ristevska-Dimitrovska et al., 2015; Liu et al., 2018). Similarly, adults with good education and social skills were more adaptable and resilient in the face of the general distress of the Great Depression and thus overcome its negative effects (Myklebust, 2000; Elder, 2001). In contrast, persons with low educational attainment have a higher risk of falling into depression when they fall victims of social events (e.g., Hurricane Katrina and the Gulf of Mexico oil spill). Psychological resilience is an important factor in reducing depression (Blackmon et al., 2016): according to a survey of survivors of the Japan earthquake and tsunami nuclear disaster, psychological resilience had an important moderating effect on depression and promotes post-traumatic growth (Kukihara et al., 2014). Similarly, in a longitudinal study of left-behind children in China, psychological resilience was shown to lessen the incidence of depression (Wu Y. et al., 2015).

Based on the above evidence, we offer the following hypothesis:

H2a Psychological resilience is negatively correlated with depression.

Evidences suggest that psychological resilience is a significant moderator against depression brought on by stressors (Luthar and Cicchetti, 2000). In the case of COVID-19, a global disaster, the depressive emotion is evident in the population, with a high prevalence of depression in college students and a moderate negative correlation between psychological resilience and depression (Taheri-Kharameh and Hazavehei, 2017; Karasar and Canli, 2020). Psychological resilience may be able to play an important role in human readjustment in the post-epidemic era, which can help individuals deal with depression, anxiety and other mood disorders that affect mental health (Osofsky et al., 2011; Shenesey and Langhinrichsen-Rohling, 2015). Therefore, it may enable them to solve everyday problems, to readjust to society and to create new opportunities (little-c). At work, for example, resilience can provide individuals with an internal mechanism to adapt, persist, and transcend, allowing them to maintain creativity through steady effort (Luthans et al., 2007). During learning, psychological resilience can provide learners with a powerful ability to challenge their environment, thereby eliciting positive emotions and motivation (Fredrickson et al., 2008; Chen and Padilla, 2019; Li and Jiang, 2020). Based on this evidence, we hypothesize that:

H2b Psychological resilience is positively correlated with creativity.

In summary, the second hypothesis is proposed:

H2 Psychological resilience plays a mediating role in the effect of depression on creativity.

### Rumination

Rumination is a steady, ongoing, recurrent metacognitive process. During the process, individuals reflect on the meaning of negative emotions (Donaldson, 2005), including thoughts about the past, present and future (Papageorgiou and Wells, 2003a). With the help of Response Style Theory, it is known that males are more likely to adopt both problem-solving and attention-shifting responses, while females are more likely to adopt ruminative thinking (Smith and Alloy, 2009). It has been argued that rumination has a negative connotation, causing people to dwell on negative emotions rather than taking more effective problem-solving measures (Teismann et al., 2014). Individual’s metacognition of ruminative thinking is the source of his or her negative emotions, i.e., the perceptions that individuals hold about ruminative thinking led to their own negative emotions (Conway et al., 2000). Excessive rumination may create a barrier to communication with others and suppress positive behaviors (Jain et al., 2007; Gebhardt et al., 2010). The process of rumination may be related to an individual’s cognitive style, causing different individuals to have different attribution patterns and ways of coping with life (Calvete et al., 2015).

Rumination can be classified as of two main types: intrusive and deliberate (Cann et al., 2011). Intrusive rumination refers to unwelcome painful reflections that are automatic intrusions of negative emotions that cause deeper suffering. Deliberate rumination is more controlled: the individual takes an active role in the construction of meaning related to the stressor, problem-solving to reach understanding, thus potentially moderating depressed emotions (Watkins, 2008; Cann et al., 2010). Deliberate rumination can affect an individual’s post-traumatic growth in response to a stressful event (Wu X. et al., 2015; Zhang et al., 2018). However, there is also evidence that the distinction between intrusive and deliberate rumination may be of little importance (Cann et al., 2011), and that the focus should be on rumination as a way of thinking and as a function (Papageorgiou and Wells, 2003b; Jain et al., 2007) rather than on its negative content (Verhaeghen et al., 2005). Nolen-Hoeksema argues that depressive states are more persistent and severe in individuals who are under ruminative thinking (Nolen-Hoeksema, 1991; Nolen-Hoeksema et al., 1994). Overall, it is clear that there is a strong association between depression and rumination. Based on this, we can formulate the following hypothesis:

H3a Deliberate rumination is correlated with depression.

There are two dimensions of rumination, including reflection on emotions and the use of problem-solving strategies. It has been showed that rumination on problem-solving is positively correlated with creativity (Vahle-Hinz et al., 2017). Rumination allows individuals to generate ideas that can remain in consciousness for a long period of time, and the indecision may contribute to creativity (Cohen and Ferrari, 2010). Therefore, we hypothesize that:

H3b Deliberate rumination is correlated with creativity.

Rumination may also be served as a mediator or moderator in depression-related research. For example, rumination played a moderating role in the effect of neurotism on the relationship between depression and anxiety (Roelofs et al., 2008). In surveys of college students, rumination was found to moderate the correlation between temperament and depression (Liu et al., 2017) and mediate the relationship between social phobia and depression (Li et al., 2019). What is more, rumination explained and regulated gender differences in depression (Jose and Brown, 2007) and played a mediating role in the relationship between depression and gender (Treynor et al., 2003). A survey of victims of cyberviolence showed that deliberate rumination partially mediated the relationship between their experiences and depression (Liu et al., 2020). Moreover, a study which was to investigate relationship between stressful life events and sleep quality have found that rumination played a mediating role and psychological resilience played a mediating role, and both of them could moderate the effects of stress on sleep quality (Li et al., 2019). Therefore, the following hypotheses can be proposed:

H3c Deliberate rumination may affect an individual’s psychological resilience.

H3d Deliberate rumination may affect the relationship between the depression and psychological resilience of an individual.

Thus, we can distill from numerous studies that rumination generally acts as a mediator or moderator between a stressor and negative emotions (e.g., anxiety and depression). Rumination also has the potential to influence an individual’s construction of emotional meaning, i.e., their psychological resilience, thereby influencing the transition from depression to post-traumatic growth. In summary, we propose the following hypothesis:

H3 Deliberate rumination may moderate the effects of psychological resilience on the relationship between depression and creativity.

## Materials and Methods

### Participants and Procedures

This study was conducted at a polytechnic college in Guangdong Province, China, that has 13 teaching units, 44 undergraduate majors and more than 20,000 students, which was formally conducted between April 10 and June 15, 2020. A total of 918 persons completed the study questionnaire, of which 881 responses were valid. Among the respondents, 317 (36.0%) were male and 564 (64.0%) were female, all of whom were from the same grade.

The value of our research was confirmed when, prior to data collection, we learned from interviews that during the COVID-19 pandemic, respondents experienced varying degrees of depression. Many interviewees said that the progress of the pandemic had made them feel uneasy and that their studies and lives had been greatly affected.

### Data Collection

This study was based on a correlational design, whereby data was collected by survey questionnaire. Between April 10 and June 15, 2020, students completed the survey by scanning QR codes during breaks in their college English classes. A QR code is a black-and-white graphic recording of data symbol information with a specific geometric pattern distributed regularly on a two-dimensional plane (Tan et al., 2018), which is widely used and accepted in China for activities such as online payment, daily travel and data entry. Participants completed the questionnaire voluntarily.

The questionnaire consisted of five sections: (a) Demographic Information, (b) Depression Scale, (c) Runco Ideational Behavior Scale, (d) Psychological Resilience Scale, and (e) Deliberate Rumination Scale. The demographic information included gender, home address and major. The scales in the questionnaire were originally designed in English, and so back-translation was used to improve the accuracy of the questions: the researcher translated the questionnaire from English to Chinese, then another researcher retranslated the Chinese version into English, and finally a third researcher compared the two versions and adjusted the scales to eliminate discrepancies.

### Measures

#### Depression Scale

Depression was measured by the Depression Anxiety Stress Scales (DASS-21), which consists of seven items created by Lovibond and Lovibond (1995) and modified by Antony et al. (1998). DASS-21 is scored on a 4-point scale that assesses the participant’s various induced negative affective states. The criteria are “1” for did not apply to me at all, “2” for applied to me to some degree, “3” for applied to me to a considerable degree and “4” for applied to me very much, with higher scores indicating a higher level of depression. In the present study, the Cronbach’s alpha coefficient for DASS-21 was 0.882.

#### Runco Ideational Behavior Scale

Creativity was assessed by the Runco Ideational Behavior Scale (Runco et al., 2001; Plucker et al., 2010; Tsai, 2015), which has 23 items. The scale focuses on individuals’ self-report level of creative behavior from everyday life, using a 5-point scale where “1” is strongly disagree, “2” is mostly disagree, “3” is uncertain, “4” is mostly agree and “5” is strongly agree. In the present study, the Cronbach’s alpha coefficient for the Runco Ideational Behavior Scale was 0.938.

#### Psychological Resilience Scale

Psychological resilience was measured by the Psychological Resilience Scale, which has 27 items (Connor and Davidson, 2003) divided into five dimensions: goal focus, emotional control, positive cognition, family support, and interpersonal assistance (Hu and Gan, 2008). The questionnaire is scored on a 5-point scale that assesses the participant’s feelings about, reactions to and agreement with the indicators. The criteria are “1” for completely inconsistent, “2” for relatively inconsistent, “3” for ambiguous, “4” for relatively consistent and “5” for completely consistent. In the present study, the Cronbach’s alpha coefficient for the Psychological Resilience Scale was 0.860.

#### Deliberate Rumination Scale

Deliberate rumination was assessed by the Rumination Scale (Cann et al., 2011), which is divided into two dimensions: intrusive rumination and deliberate rumination (Cann et al., 2011). In this study, the deliberate rumination section of the Rumination Scale was selected, which consists of 10 items and is scored on a 4-point scale, with “1” for not at all, “2” for occasionally, “3” for frequently and “4 “for always. In the present study, the Cronbach’s alpha coefficient for the deliberate rumination section of the Rumination Scale was 0.913.

### Statistical Analyses

The analysis tool used in this study was IBM SPSS 23.0.

First, we performed Harman’s single factor test to examine common method bias (Podsakoff et al., 2003). The results of unrotated principal component analysis showed that 21 factors had eigenvalues greater than 1, of which the contribution to the total variance was 65.811%. The first factor accounted for only 17.830%, which is far below the critical criterion of 40% (Li, 2018), indicating that there was no significant common method bias. In other words, the variation between the independent and dependent variables was caused more by difference in the nature of variables than by the methods of data collection and measurement.

Following the test of common method bias, descriptive statistical analysis was performed: the mean and standard deviation of each variable were calculated to observe the trend of concentration and dispersion. Then, Pearson product-moment correlation coefficients were calculated to test the correlation on all variables (see Table 1).

**TABLE 1:** Means, standard deviations, and correlations among variables.

	***M***	***SD***	**(1)**	**(2)**	**(3)**	**(4)**	**(5)**
(1) Gender	1.640	0.480	–				
(2) Depression	1.635	0.568	−0.140**	–			
(3) Creativity	3.249	0.563	–0.035	0.085*	–		
(4) PR	3.417	0.443	−0.076*	−0.462**	0.198**	–	
(5) DR	2.012	0.520	0.071*	0.138**	0.288*	0.078*	–

*p < 0.05, **p < 0.01, ***p < 0.001. PR, psychological resilience; DR, deliberate rumination.

Finally, PROCESS V3.3 (Hayes, 2013) was used to test the mediation model (Model 4) and the moderating mediation model (Model 7). For both models gender was used as a control variable. Indicators of indirect effects were tested using a bias-corrected bootstrapping (*n* = 5,000) with 95% confidence intervals. When the 95% confidence interval does not include zero, it indicates that the parameter is statistically significant (Shrout and Bolger, 2002).

## Results

### Descriptive Statistics and Correlation Analysis

Descriptive analyses and correlations among variables are shown in Table 1.

As expected, depression and creativity were positively and weakly correlated in college students (*r* = 0.085, *p* < 0.05); depression and psychological resilience were significantly and negatively correlated (*r* = −0.462, *p* < 0.01); psychological resilience was significantly and positively correlated with creativity (*r* = 0.198, *p* < 0.01); deliberate rumination was positively correlated with depression (*r* = 0.138, *p* < 0.01); deliberate rumination showed a positive correlation with psychological resilience (*r* = 0.078, *p* < 0.05); and deliberate rumination showed a positive correlation with creativity (*r* = 0.288, *p* < 0.05). Therefore, the correlation of study variables aligned with the hypotheses.

### Mediating Effect of Psychological Resilience

Mediation analysis was carried out using depression as the independent variable, creativity as the dependent variable and psychological resilience as the mediator.

Since gender may affect variables, it was used as a control variable. Gender was transformed into a dummy variable before entering the mediation model. When gender was controlled, the correlation coefficient between variables changed very little, so it had little effect on the overall mediation model.

As is shown in Table 2, the total effect of depression on creativity was statistically significant (β = 0.219, *SE* = 0.037, *P* < 0.001), indicating that an appropriate depressive mood had a positive predictive effect on creativity. This predictive effect remained significant when psychological resilience was added (β = −0.138, *SE* = 0.020, 95% CI = [−0.180, −0.100]). Depression had a significant negative effect on psychological resilience (β = −0.359, *SE* = 0.024, *P* < 0.001), and psychological resilience had a significant positive effect on creativity (β = 0.384, *SE* = 0.046, *P* < 0.001). Psychological resilience mediated the model through its negative association with depression.

**TABLE 2:** Testing the mediating effect of depression on creativity.

**Preditors**	**On psychological resilience**					**On creativity**			
	**β**	**SE**	**t**	**95%CI**		**β**	**SE**	**t**	**95%CI**
Gender	0.011	0.028	0.385	[−0.044, 0.066]		–0.032	0.038	–0.840	[−0.108, 0.043]
Depression	–0.359	0.024	−15.216***	[−0.406, −0.31]		0.219	0.037	5.994***	[0.147, 0.291]
PR	–	–		–	–	0.384	0.046	8.264***	[0.291, 0.475]
R2	0.213	–		–	–	0.080	–		–
F	118.985	–		–	–	25.235	–		–
			
Direct effect of X on Y	**β**	**SE**	**t**	**95% LLCI**	Indirect effect (s) of X on Y	**β**		**SE**	**95% BootCI**
			
	0.219	0.037	5.994***	[0.147, 0.291]		–0.138		0.020	[−0.180, −0.100]

*p < 0.05, **p < 0.01, ***p < 0.001. PR, psychological resilience. Analyses conducted by PROCESS Model 4, N = 881. Gender was dummy coded (1, female, 0, male).

In addition, bias-corrected bootstrapping revealed further moderating mediation effects, with confidence intervals (95%) that did not include zero values between the upper and lower bounds (see Table 2). This suggests that depression can have a direct effect on creativity as well as an indirect effect on creativity through psychological resilience, with direct effects accounting for 37.052% of the total effect and mediating effects for 62.948%, respectively.

### Moderated Mediation Effects

Gender also served as a control variable in the mediation model test conducted by PROCESS model7.

**FIGURE 1:**
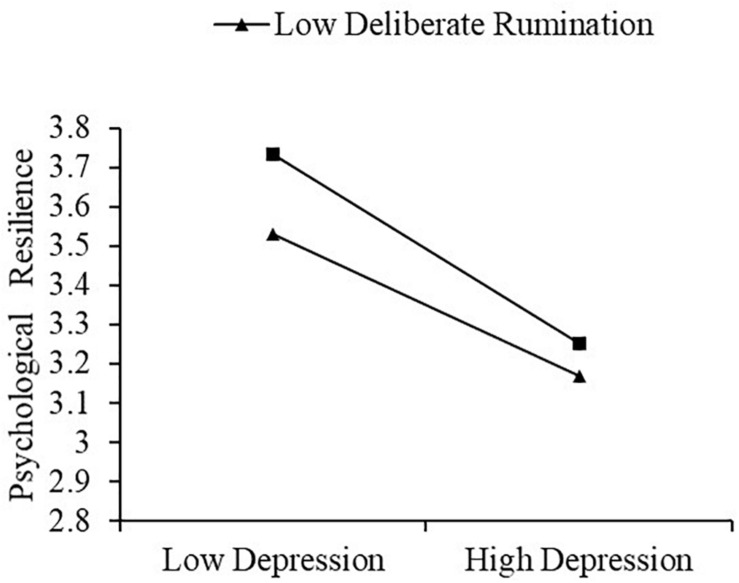
Plot of the interaction between depression and deliberate rumination on psychological resilience.

Table 3 shows that had a significant moderating effect on psychological resilience (β = −0.104, *t* = −2.863, *P* < 0.01). To better test the moderated mediation model, the scores for deliberate rumination were divided into high (1 standard deviation above the median), medium and low groups (1 standard deviation below the median) (Table 4), and a simple slope test was performed (Figure 1). The results indicated (Figure 1) that depression combined with high deliberate rumination (β = −0.426, *SE* = 0.029, *p* < 0.001, 95% CI = [−0.484, −0.369]) was more strongly associated with psychological resilience than depression combined with low deliberate rumination (β = −0.318, *SE* = 0.031, *p* < 0.001, 95% CI = [−0.379, −0.257]).

**TABLE 3:** Examination of the moderating effect of depression on creativity.

**Preditors**	**On psychological resilience**				**On creativity**			
	**β**	**SE**	**t**	**95%CI**	**β**	**SE**	**t**	**95%CI**
Gender	–0.005	0.028	–0.171	[−0.059, 0.049]	–0.032	0.038	–0.084	[−0.108, 0.043]
Depression	–0.372	0.024	−15.81***	[−0.418, −0.326]	0.219	0.037	5.994***	[0.147, 0.291]
DR	0.136	0.026	5.269***	[0.086, 0.187]				
PR					0.384	0.046	8.264***	[0.292, 0.475]
Depression × DR	–0.104	0.036	−2.863**	[−0.176, −0.033]				
R2	0.241	−	−	−	0.079	−		−
F	69.398	−	−	−	25.235	−		−

Analyses conducted using PROCESS model 7 with N = 881. Gender is dummy coded (1, female, 0, male). PR, psychological resilience; DR, deliberate rumination. p < 0.05, **p < 0.01, ***p < 0.001.

**TABLE 4:** Results for conditional indirect effect of depression on creativity via PR across levels of deliberate rumination.

**Moderator**	**Level**	**Effect size**	**Boot SE**	**LL 95% CI**	**UL 95% CI**
Deliberate rumination	Low (M−1SD)	−0.318	0.031	−0.379	−0.257
	Medium (M)	−0.372	0.024	−0.418	−0.326
	High (M + 1SD)	−0.426	0.029	−0.484	−0.369

*Bootstrap sample size = 5,000. Low, 1 SD below the mean. High, 1 SD above the mean. PR, psychological resilience; DR, deliberate rumination.*

**FIGURE 2:**
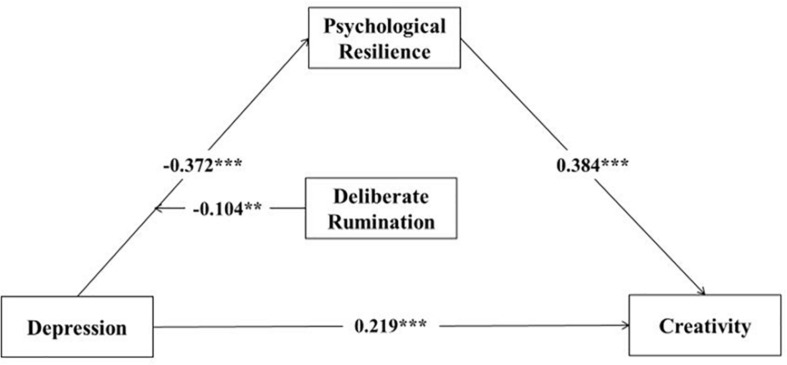
The moderated mediation model that controls for gender. *p < 0.05; **p < 0.01; ***p < 0.001.

Figure 2 shows the moderated mediation model, in which deliberate rumination moderated the effects of depression on psychological resilience, and deliberate rumination regulates just the first stage of the mediation process.

## Discussion

### Discussion of Results

In this study, we developed a moderated mediation model of the relationship between depression and creativity among college students during the pandemic of COVID-19. It was found that: (1) depression is positively correlated with creativity; (2) the positive association between depression and creativity is partially mediated by psychological resilience; and (3) deliberate rumination is able to play a moderating role in the direct effect of depression on creativity. These results are consistent with the proposed hypotheses and previous findings.

First, the results were consistent with H1 and the existing literature, revealing that depression is positively correlated with creativity. This finding indicates that depression does not just generate negative effects but also may prompt an individual to be more creative (De Dreu et al., 2008; Nijstad et al., 2010). During the quarantine of COVID-19 outbreak, individuals’ physical activities were significantly reduced (Khosravi, 2020), while research has proven that if regular physical activity is performed in a confined environment, it can prevent and alleviate individual’s depression and anxiety (Takács, 2014). Therefore, the lack of physical activity during the confinement may exacerbate depression and anxiety (Ingram et al., 2020; Lopez et al., 2020). And when a person is of depression, it may affect the one’s ability to cope with life events, thus affecting daily creativity (little-c). The dual pathway to creativity model developed by De Dreu et al. (2008), suggests that mood states primarily influence creativity through cognitive flexibility and cognitive persistence (De Dreu et al., 2008; Nijstad et al., 2010): activating negative moods improves cognitive persistence, making it possible to better perform analytical tasks (Huntsinger and Ray, 2016). These findings are consistent with the cognitive tuning theory, which suggests that the bar for problem-solving effectiveness may be set higher when people are in a negative mood state than when they are in a positive mood state (Schwarz, 2000). An individual in negative affect may prolong the process of gathering and processing problem-relevant information, thereby enhancing creativity (Fredrickson and Branigan, 2005; Lin et al., 2013). Similarly, according to the cognitive model of depression, depressive thinking is related to bottom-up cognitive processing (Disner et al., 2011; Kwak et al., 2016), which enables negative emotions to generate powerful self-reflective thoughts and perseverance, leading to creative performances (Akinola and Mendes, 2008; De Dreu et al., 2008).

The results of the present study also validated H2. First, the results showed that depression negatively predicts psychological resilience, which is consistent with previous studies (Karasar and Canli, 2020) revealing that strong psychological resilience may alleviate depression (Taheri-Kharameh and Hazavehei, 2017). In other words, psychological resilience has been shown to have an important adaptive, regulatory function in recovery from depression (Li et al., 2015; Wojujutari et al., 2019) by allowing individuals’ resistance and self-protection to come into play (Rutter, 2018). As well, greater psychological resilience can stimulate positive emotions in individuals that enable them to emerge from pain (Ong et al., 2010). Second, psychological resilience is a positive predictor of creativity, which is consistent with psychological and sociological findings that when individuals have strong psychological resilience, their problem-solving stamina and resilience are at a higher level, thus promoting creativity. Creativity is associated with the social component of psychological resilience because creative activity, in turn, psychological resilience is supported by the social environment and relationships that reinforce participation in creative activity, which means that creative social engagement and psychological resilience are mutually supportive (Hertzog et al., 2008; McFadden and Basting, 2010).

Furthermore, we found that psychological resilience can act as a mediator between depression and creativity, which supports H2. Neurologically, psychological resilience appears to be related to emotional processing (Shi et al., 2019). More precisely, psychological resilience is associated with functional connectivity between the left orbitofrontal gyrus and the left inferior frontal gyrus of the brain, which flexibly use emotional resources and control interest processing (Shi et al., 2019). Thus, individual with high resilience may be able to capitalize on their emotional resources in the face of adversity or threat (Debbane et al., 2017), enabling them to survive and thrive as they adjust positively to adversity (Sweetman et al., 2011). Therefore, it can be inferred that psychological resilience may play an important role in re-adaptation during the post-pandemic era, helping individuals deal with depression, anxiety and other mood disorders that affect mental health (Osofsky et al., 2011; Shenesey and Langhinrichsen-Rohling, 2015). Psychological resilience may also prompt individuals to find better solutions to problems of daily life and readjustment to society, creating new opportunities (little-c).

Finally, our findings are consistent with H3, revealing a correlation between deliberate rumination and depression, psychological resilience and creativity, suggesting that deliberate rumination plays a moderating role in the mediation model. The study results show that deliberate rumination moderates the effects of psychological resilience on the relationship between depression and creativity, which echoes the findings of previous studies (Rider Mundey et al., 2018; Kim and Bae, 2019). The implication is that, when individuals are in depression, those who have high levels of deliberate rumination may analyze depression metacognitively, generating powerful self-reflection and perseverance (Akinola and Mendes, 2008; De Dreu et al., 2008). This process may turn negative emotions into positive constructs of meaning to help individuals achieve post-traumatic growth, thereby increasing their psychological resilience (Solem et al., 2016). In reality, after a longer period of depressed emotional state, using psychological resilience as a mediating path, college students are often able to emerge from the trauma and grow. At the same time, deliberate rumination further strengthens psychological resilience, which ultimately acts on individual’s creativity.

### Discussion of Implications

From a theoretical perspective, this study establishes a link between depression and creativity, thus deepening the discussion of the effect of depression on creativity and enhancing related research findings. In addition, we have found that psychological resilience mediates the effects of depression on creativity and that, in turn. deliberate rumination moderates the mediating effect of psychological resilience. This finding implies that college students suffering from depression may enhance their ability to adjust positively and to cope with challenges by increasing their psychological resilience, which could allow them to express their creative potential in their daily life or in their areas of expertise.

From a practical perspective, the relationship revealed between the four variables by this study may help researchers better understand the mechanisms by which depression stimulates creativity, thus providing different entry points for emotional healing and psychological guidance.

### Limitations and Future Direction

The limitations of this study are apparent. First, the study is a cross-sectional study, so not enough has been done on longitudinal studies. Second, all participants were from the same university, and the group comprised mainly college students, therefore, the sample of this study is not universally representative and the results of this study cannot be generalized. This study is only used as an independent research study for reference and does not have the significance of generalizing the results.

Future researchers might carry out longitudinal research to follow up with participants. In addition, researchers could explore which other mediating variables may frame the effects of depression on creativity and whether the effects of depression differ according to dimensions of creativity.

The mechanisms and cognitive processes associated with depression and its effects on creativity are still contested, but this study may provide empirical evidence and insights for future researchers.

## Conclusion

This study explored the relationship between depression and creativity, the mediating effect of psychological resilience between the two and the moderating effect of deliberate rumination on psychological resilience. The results suggest that depression is a positive predictor of creativity and may promote creativity to some extent. Further, individuals with greater psychological resilience are more creative than those with less psychological resilience, as it is a question of whether they can and to what extent they can effectively use depression as an emotional resource. Last, an individual’s level of deliberate rumination moderates the mediating process, especially at the stage where depression is associated with psychological resilience. These findings advance understanding of the mechanisms that operate between depression and creativity.

## Data Availability Statement

The raw data supporting the conclusions of this article will be made available by the authors, without undue reservation.

## Ethics Statement

The studies involving human participants were reviewed and approved by the Institutional Ethics Committee of School of Geography, South China Normal University. The patients/participants provided their written informed consent to participate in this study.

## Author Contributions

YX and WZ designed the research and reviewed and edited the manuscript. JS, YX, WZ, and DH reviewed the literature and analyzed the data. YX, JS, WZ, YZ, XW, JW, and DH wrote the manuscript. All authors have read and agreed to the published version of the manuscript.

## Conflict of Interest

The authors declare that the research was conducted in the absence of any commercial or financial relationships that could be construed as a potential conflict of interest.
